# Ameloblastic fibrodentinosarcoma: a rare malignant odontogenic tumor^☆^

**DOI:** 10.1016/j.bjorl.2015.04.009

**Published:** 2015-09-08

**Authors:** Leorik Pereira da Silva, Jefferson da Rocha Tenório, Bartolomeu Cavalcanti de Melo Júnior, José Paulo da Silva Filho, George João Ferreira do Nascimento, Ana Paula Veras Sobral

**Affiliations:** a Universidade Federal do Rio Grande do Norte (UFRN), Departamento de Odontologia, Patologia Oral, Natal, RN, Brazil; b Hospital Universitário Oswaldo Cruz, Departamento de Cirurgia de Cabeça e Pescoço, Recife, PE, Brazil; c Instituto São Leopoldo Mandic, Centro de Pesquisas, SLMandic, São Paulo, SP, Brazil; d Universidade Federal de Campina Grande (UFCG), Faculdade de Odontologia, Centro Acadêmico de Ciências Biológicas, Patologia Oral, Patos, PB, Brazil; e Universidade de Pernambuco (UPE), Faculdade de Odontologia, Patologia Oral, Camaragibe, PE, Brazil

## Introduction

Ameloblastic fibrodentinosarcoma (AFDS) is a rare odontogenic tumor histologically characterized by a sarcomatous ectomesenchymal component associated with variable amounts of benign ameloblastomatous epithelium and the presence of dysplastic dentin.^1^ About 14 cases have been reported in the medical literature. The etiology of the AFDS is poorly understood; however, approximately one-third of AFDS appear to represent the malignant transformation of pre-existing ameloblastic fibrodentinoma.^1^, ^2^

AFDS has a predilection for the mandible, and it is most commonly seen in male patients in the third decade of life. Patients often present with a painful swelling, and AFDS is radiographically characterized by a multilocular radiolucent lesion with indistinct margins, with or without radiopaque foci. Metastases are rare, but recurrences have been reported. The treatment of choice is wide surgical resection.^3^

Given the above, the aim of this report is to describe a case of aggressive mandibular AFDS with emphasis on clinical, radiological, and histopathological aspects.

## Case report

**Figure 1:**
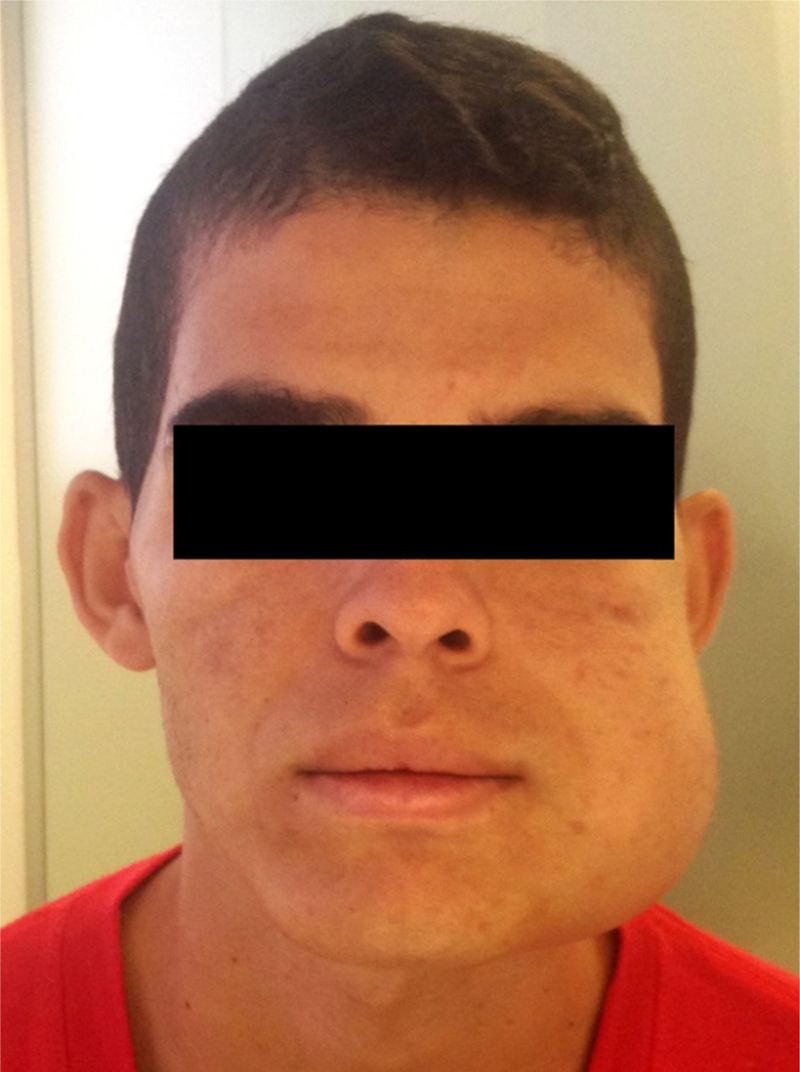
Clinical findings. Front view of the patient showing marked facial asymmetry.

A 19-year-old man was referred to public service for oral pathology consultation in 2014. The extraoral inspection showed a large swelling with strong facial asymmetry on left side of the face (Fig. 1). Intraorally, an extensive ulcerated and necrotic mass was observed from the tooth 35 extending to the mandible body and ramus, with clinical absence of teeth 36, 36, and 38. Despite the presence of ulceration, the patient only reported pain on palpation, and limitation in mouth opening was noted. There was no clinical evidence of regional lymphadenopathy.

**Figure 2:**
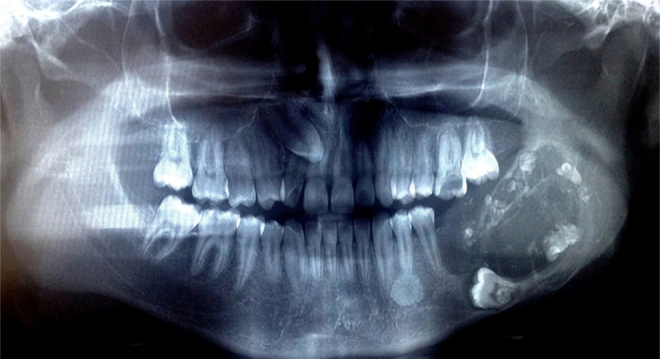
Panoramic radiograph showing multilocular extensive radiolucent lesion with radiopaque foci in the left mandible, associated with impacted molar (36).

The radiographic exam revealed an ill-defined radiolucency measuring about 7 cm × 4.5 cm associated with impacted molar (36), lacking sclerotic borders and associated with the presence of multiple radiopaque flecks. Expansion and thinning of cortical bone were also observed (Fig. 2). Calcifying epithelial odontogenic tumor, calcifying odontogenic cyst, and ameloblastic fibrodontoma were the main clinical differential diagnoses considered.

**Figure 3:**
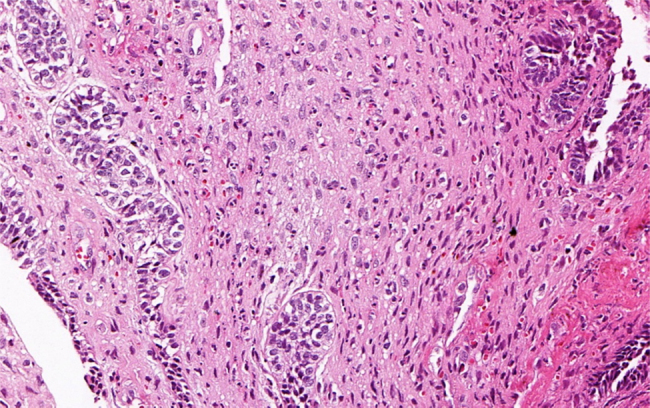
Histopathological findings. Biphasic pattern with benign odontogenic epithelium surrounded by sarcomatous component with intense cellular and nuclear pleomorphism and mitotic figures (H&E stain, 200×).

**Figure 4:**
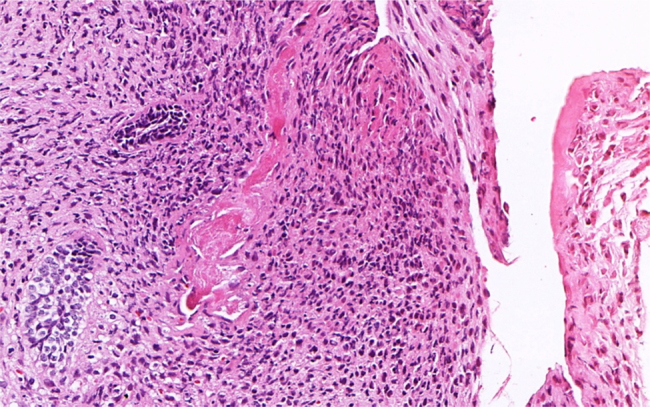
Histopathological aspects showing deposition of dentinoid-like material. (H&E stain, 200×).

An incisional biopsy was performed and submitted to histopathological exam. Microscopic examination of the sections routinely stained in hematoxylin and eosin revealed a biphasic neoplastic proliferation of odontogenic epithelial and mesenchymal tissue. The odontogenic epithelial component consisted of multiple cords and islands bounded by columnar to cuboidal ameloblast-like cells, with reversed nuclear polarity. In the center of these structures, the neoplastic cells had a loose aspect, resembling the stellate reticulum of the enamel organ. The mesenchymal component consisted of a primitive connective tissue that displayed marked pleomorphism characterized by variation in cell size and shape, as well as nuclear hyperchromatism, alteration in nuclear to cytoplasmic ratio, and scattered mitotic figures (Fig. 3). Areas of necrosis focus were identified. Juxta-epithelial hyalinization was evident in few areas. Focal areas of dentinoid-like material with tubules were also observed (Fig. 4). Enamel formation could not be identified, even on multiple sections. Furthermore, ameloblastic fibroma areas were present. Therefore, clinical, radiographic, and histopathological findings supported the diagnosis of ameloblastic fibrodentinosarcoma.

The oncologist and surgeon chose to perform chemotherapy previous to the surgical treatment. Initially, three cycles of a drug combination were administered, comprising vincristine, cyclophosphamide, and cloxorubicin. The patient did not respond well to initial treatment and did not appear for the surgery on the day scheduled, returning only after four weeks. Thus, a new combination of drugs in three cycles was instituted, consisting of ifosfamide, carboplatin, and etoposide in order to reduce tumor size before surgery.

**Figure 5:**
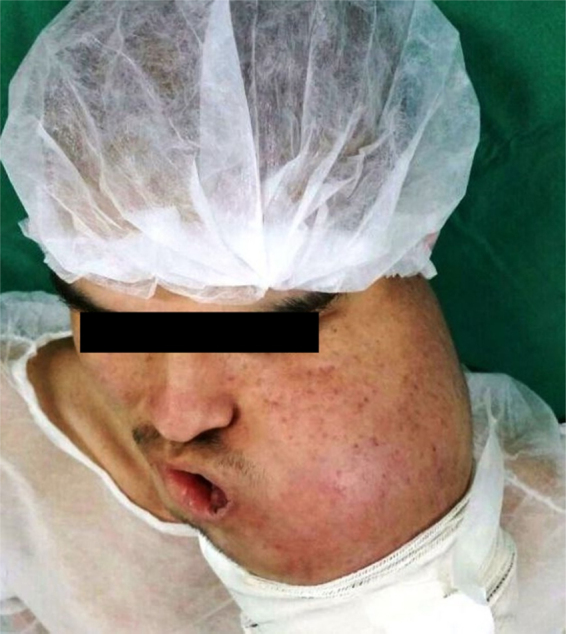
Craniocaudal view of the patient showing a significant increase in tumor mass after eight sessions of chemotherapy.

However, after two cycles of chemotherapy with different drugs, the patient showed a significant increase in tumor mass with skin invasion, limited mouth opening, and extreme facial deformity (Fig. 5). After the unexpected aggressive evolution of the tumor, surgeons performed radical left hemimandibulectomy with peripheral myotomy of the muscle insertions associated with resection of the affected skin. The bone margin of 3 cm before the mandibular symphysis and the peripheral muscles removed with a margin of 2 cm were tumor-free. The left inferior alveolar nerve was involved by the tumor; however, there was no presence of invasion of blood or lymphatic vessels. Ipsilateral supraomohyoid neck dissection of 18 lymph nodes showed no tumor cells. The patient underwent postoperative radiotherapy with 6000 Gy.

The patient has been re-evaluated every three months with chest X-ray and CT scan of the head and neck. There has been no evidence of regional or distant metastasis, and the patient has been under clinical follow-up for one and a half years.

## Discussion

Odontogenic tumors (OT) consist of a group of rare and heterogeneous lesions, representing less than 4% of all specimens of the oral and maxillofacial region. Malignant odontogenic neoplasms are even rarer and constitute a small percentage of OT. In various series published worldwide, the frequency varies between 0% and 6.1%.^4^ The pathogenesis of malignant OT is unclear, although some authors have suggested alterations in cellular cycle, expression of proto-oncogenes, and mutations in tumor suppressor genes in the pathogenesis of these lesions.^4^

Microscopically, the bland epithelial component of AFDS is similar to that seen in ameloblastic fibrosarcoma, although it is less frequent.^5^, ^6^ The definitive diagnosis of AFDS is established based on histopathologic evaluation of the mesenchymal component, which usually demonstrates features of malignancy, including cellular atypia, pleomorphism, and mitotic figures. In addition, when a material similar to the dentinoid is observed, the final diagnosis should be AFDS.^7^, ^8^

Despite the morphological differences, the World Health Organization (WHO) distinguishes odontogenic sarcoma devoid of dental hard tissue (ameloblastic fibrosarcoma) from those displaying focal evidence of dentinoid (ameloblastic fibrodentinosarcoma) or dentinoid plus enameloid (ameloblastic fibro-odontosarcoma), but the WHO panel acknowledges that presence or absence of dental hard tissue in an odontogenic sarcoma is of no prognostic significance. The literature reports that the biological behavior of the AFDS is, in general, similar to other odontogenic sarcomas, with high local aggressiveness and low potential for regional lymph node involvement or distant metastasis.^6^, ^7^, ^8^

Radiographically, AFDS can show a uni- or multilocular appearance, with poorly circumscribed outlines associated with tooth and one or more dense opacities. The present case showed a multilocular appearance associated with the left lower first molar (36) and slight dense opacities. These radiographic findings and the location of the mass highly suggest the possibility of odontogenic cysts and tumor.^3^, ^8^ The clinico-radiographic differential diagnosis should include calcifying epithelial odontogenic tumor, calcifying odontogenic cyst, and ameloblastic fibrodontoma. However, a case with an irregular border and expansion and perforation of the cortexes should be interpreted with caution, and the possibility of malignant odontogenic tumor should be suspected.

The mean age at the time of diagnosis of AFDS in 62 cases reviewed by Bregni et al.^7^ was 27.3 in a wide age range, from 3 to 83 years. According to these 62 published cases, the tumor is more common in males than females (59.7% *vs.* 37.1%). Furthermore, odontogenic sarcomas are more frequent in the mandible (79% of cases) than the maxilla (21%), with the majority of the cases located in posterior region of the mandible.^9^

Odontogenic sarcomas are reported as a highly recurrent lesion. Till date, 25 (35%) of the 71 reported cases have had at least one recurrence during follow-up period and 14 patients (19.7%) have died of their disease within three months to 19 years. Clinical findings vary among reported cases, but usually signs and symptoms include pain and swelling. Despite regional lymph node involvement or distant metastases reported in few cases, some authors have considered AFDS as a low-grade sarcoma.^2^, ^3^, ^4^, ^5^, ^6^, ^7^, ^8^

In a recent multicenter epidemiological study conducted in Latin America, 25 cases of malignant odontogenic tumors were reported, and six cases were odontogenic sarcomas. All cases had the diagnosis of ameloblastic fibrosarcoma and no case of AFDS was identified.^10^ In addition, in a retrospective study conducted in Brazil, 240 odontogenic tumors were described; however, no cases of odontogenic sarcoma were found.^11^

The treatment of choice for AFDS is radical surgical excision without primary neck dissection. Some investigators recommend adjuvant chemotherapy and/or radiotherapy, but its benefits are uncertain.^3^ Owing to the rarity of cases, it is difficult to accurately estimate long-term prognosis. Particularly in this case, the patient did not satisfactorily respond to chemotherapy before surgery, leading to clinical worsening and increased tumor aggressiveness; this fact emphasizes the dilemma of whether or not the adjuvant chemotherapy should be indicated in the treatment of these sarcomas.

## Conclusion

In summary, this case report describes the first case of AFDS in Brazil and emphasizes the importance of considering the odontogenic sarcomas as a differential diagnosis of maxillary osteolytic lesions, despite being extremely rare lesions. Thus, these lesions are a challenging diagnosis for clinicians and pathologists.

## Conflicts of interest

The authors declare no conflicts of interest.
